# *Gentiana straminea* supplementation improves feed intake, nitrogen and energy utilization, and methane emission of Simmental calves in northwest China

**DOI:** 10.5713/ab.21.0263

**Published:** 2021-10-29

**Authors:** K. L. Xie, Z. F. Wang, Y. R. Guo, C. Zhang, W. H. Zhu, F. J. Hou

**Affiliations:** 1 State Key Laboratory of Grassland Agro-ecosystems, Key Laboratory of Grassland Livestock Industry Innovation Ministry of Agriculture, College of Pastoral Agriculture Science and Technology, Lanzhou University, 730000, China

**Keywords:** CH_4_ Emission, Energy Utilization, *Gentiana straminea*, Natural Plants, Nitrogen Utilization

## Abstract

**Objective:**

Native plants can be used as additives to replace antibiotics to improve ruminant feed utilization and animal health. An experiment was conducted to evaluate the effects of *Gentiana straminea* (GS) on nutrient digestibility, methane emissions, and energy metabolism of Simmental calves.

**Methods:**

Thirty-two (5-week-old) male Simmental clves, with initial body weight (BW) of 155±12 kg were fed the same basal diet of concentrates (26%), alfalfa hay (37%), and oat hay (37%) and were randomly separated into four treatment groups according to the amount of GS that was added to their basal diet. The four different groups received different amounts of GS as a supplement to their basal diet during whole experiment: (0 GS) 0 mg/kg BW, the control; (100 GS) 100 mg/kg BW; (200 GS) 200 mg/kg BW; and (300 GS) 300 mg/kg BW.

**Results:**

For calves in the 200 GS and 300 GS treatment groups, there was a significant increase in dry matter (DM) intake (p<0.01), average daily gain (ADG) (p<0.05), organic matter intake (p<0.05), DM digestibility (p<0.05), neutral detergent fibre (NDF) digestibility (p<0.05), and acid detergent fibre (ADF) digestibility (p<0.05). Dietary GS supplementation result in quadratic increases of DM intake (p<0.01), ADG (p<0.05), NDF intake (p<0.05), and ADF intake (p<0.05). Supplementing the basal diet with GS significantly increased nitrogen (N) retention (p<0.001) and the ratio of retention N to N intake (p<0.001). Supplementing the basal diet with GS significantly decreased methane (CH_4_) emissions (p<0.01), CH_4_/BW^0.75^ (p<0.05) and CH_4_ energy (CH_4_-E) (p<0.05). Dietary GS supplementation result in quadratic increases of CH_4_ (p<0.01) and CH_4_/DM intake (p<0.01). Compared with 0 GS, GS-supplemented diets significantly improved their gross energy intake (p<0.05). The metabolizable energy and digestive energy intake were significantly greater for calves in the 100 GS and 200 GS calves than for 0 GS calves (p<0.05).

**Conclusion:**

From this study, we conclude that supplementing calf diets with GS could improve utilization of feed, energy, and N, and may reduce CH_4_ emissions without having any negative effects on animal health.

## INTRODUCTION

Research into CH_4_ emissions is increasing as the amount of CH_4_ in the atmosphere increases. This is because of its impact on temperature and climate change, as well as its effect on the ecological environment [1]. CH_4_ is the second most abundant anthropogenically sourced greenhouse gas and contributes 23 times as much as carbon dioxide (CO_2_) to climate warming [2]. It has been estimated that ruminant gut emissions account for 15% of total global CH_4_ emissions [3] and that 3% to 10% of the energy that ruminants receive from feed is used for CH_4_ emissions [4]. Limiting CH_4_ emissions from ruminants is therefore one way to protect the ecological environment and to make feed utilization more efficient.

Phytogenic alternatives are commonly used in place of chemical feed additives (e.g., antibiotic, antiprotozoal agents, hormones, and ionophores) in animal husbandry, because many chemical feed additives can have a detrimental effect on food quality and on the ecological environment, and their use has been restricted by the globally [5]. Native plants can grow in harsh environments such as deserts, severe cold, and high altitudes and can have abundant functional components related to secondary physiological metabolism [6]. According to the feeding preferences of animals, native plants are divided into desirable and undesirable herbage. Some of desirable native plants (e.g., mulberry leaf, *Allium Mongolicum*, and *Cistanche deserticola*) have been found to significantly enhance ruminant growth, dry matter intake (DMI), and nutrient digestibility, and in the last five years to reduce CH_4_ emissions [6–8]. Globally, ~ 60% of the herbage on rangeland is desirable [9], and up to 15% of the herbage ingested by grazing livestock is undesirable [10]. Undersirable herbage is thought to cause serious problems for livestock on pasture [11]. However, some herbage that is considered undesirable with respect to livestock grazing is also used as a Chinese herbal medicine in East Asia countries [12]. This herbage can enhance the appetite, have antibacterial and anti-inflammatory activities, and can strengthen the immune system [13]. Despite the inevitable intake of undersirable herbage while livestock graze on rangeland or sown pasture, or are fed in a pens [14], little research has carried out into its effects. Therefore, this study investigated the following hypotheses: Undesirable native plants have the opposite effect on ruminants as desirable native plants such as increasing CH_4_ emissions, reducing DMI and dry matter digestibility (DMD).

To test the hypothesis, we used *Gentiana straminea* (GS), which is widely distributed in high-altitude areas of grassland. It is rich in simple secoiridoid glycoside (6.51%), flavonoids (3.87%), and iridoid glycosides (2.28%) [12]. The roots of GS have been used in Chinese herbal medicine to ptomote digestion and as an antioxidant and antibacterial herbal that can improve the immune response [15]. GS inhibits many Gram-negative and Gram-positive bacteria, which may alter rumen fermentation as well as feed utilization among rumints [12]. In the Hexi region of China, grazing is typically used to raise livestock. GS is widely distributed on grazing grassland and is a common undesirable herbage, which cattle inevitably eat on pasture. This study aimed to i) determined the effects of supplementing the diets of calves with GS on intake, digestion, CH_4_ emissions, and N metabolism, ii) evaluate the optimal amount of GS to add to livestock diets to improve N digestibility and reduce CH_4_ emissions. This study will deepen our understanding of undesirable herbage and provide insights useful for implementing any needed mitigations.

## MATERIALS AND METHODS

### Experimental animals and design

The study site was located at the Linze Grassland Agricultural Trial Station of Lanzhou University, Linze County, Gansu Province, China. The climate is a temperate continental climate, with a mean annual temperature of 7.7°C and mean annual precipitation of 118.4 mm. The dominant agricultural systems here are intensively and extensively specialized crop and livestock production systems [16].

The animal care and experimental procedures were approved by the Animal Use and Care committee of Lanzhou University (Gansu, China, No. 2010–1 and 2010–2). The 32 male Simmental calves in the trial were all almost 5 months old, and all had a body weight (BW) of 155±12 kg. A completely randomized single-factor design was used for the 70-day study period. Calves were divided into four separate treatment groups (selection for each group was random with respect to BW). The calves were housed in individual pens and could freely access to clean water. The calves were fed a basal diet that included 260 g of mixed concentrate (10.44% of soybean meal and 15.66% of wheat bran), 370 g of alfalfa hay, and 370 g of oat hay (Table 1), all per kilogram dry matter (DM), and met their nutrient requirements in accordance with the feeding and nutritional standards for beef cattle [17]. All the animals were adapted to the basal diet prior to the experimental period. One group of calves received the basal diet with no supplements, this was the control group (0 GS). In the three treatment groups, the basal diet was supplemented with 100 mg (100 GS), 200 mg (200 GS), and 300 mg (300 GS) of GS per kilogram BW per calf per day during whole experiment. The root of GS was obtained from Gansu and provided by Lanzhou Foci Pharmaceutical Co., Ltd (Lanzhou, China). The GS was pulverized into powder and provided to calves. The amount of GS was set based on the optimal dose of 200 mg/kg BW for mice [12]. The claves were fed alfalfa hay and oat hay at 08:00 and 19:00 and mixed concentrate at 14:00 daily. The added powder of GS was included with 100 g of mixed concentrate DM in the 08:00 feed, to ensure that all the GS was eaten.

Each calf was accommodated in a separate pen and fed the basal diet and supplements for fourteen days. After this, they were moved to metabolic chambers for another five days, during which the feed amount, residual feed amount, and excreted feces and urine were recorded. Finally, they were moved to separate, indirect open-circuit respiration calorimeter chamber for a another three days, during which CH_4_ emissions were recorded. There was one calf in each chamber, and all metabolic chambers were equipped with a trough, an automatic drinking device, and separate trays to collect fecal samples; urine was collected in a plastic container with a handmade urine bag. The calves in the chambers were fed the basal diet and supplements, and all of the residual feed was collected. The 32 calves were divided into eight time periods and transferred to metabolic chamber and indirect open-circuit respiration calorimeter chambers in batches. There only four calves in each time period, as there were only four indirect open-circuit respiration calorimeter chambers. During these eight time periods, all calves were fed basic diet and supplements.

### Gas measurement and sampling

The amount of offered forage and concentrate, and the residual feed amount were recorded at feeding times every day for each calf. The collected samples were dried at a constant temperature of 65°C until they reached a constant weight, which was recorded as the DM amount. Calves were weighed on entering and leaving the chamber. The weight change over the entire feeding period was taken as the average daily gain (ADG). The metabolism chamber and the indirect open-circuit respiration calorimeter chambers are both independent, so this study used overlapping experiments. The 8 days of moving the calf into the chamber mainly included the following parts: the first day was used for acclimatization, after which the residual feed, feces, and urine were measured and analyzed for metabolic data over days 2 to 5 in the metabolism chamber, and CH_4_ emissions were measured over days 6 to 8 in the indirect open-circuit respiration calorimeter chambers. There is an overlap between the first time period and the scond time period, and the second time period is started on the 4th day of the first time periof. Other time periods are also arranged like this. Therefore, the sample and data collection period totaled 29 days. Feces were collected and weighed every two hours every day. The fresh feces collected over days 2 to 5 were combined for each calf and divided into two portions. One portion was dried at 65°C for 96 hours, for analysis of fecal energy (FE), N, fibre content, and organic matter (OM). The other portion of fresh feces (100 g) was combined with 10 mL of 10% (v/v) sulfuric acid (H_2_SO_4_) for N fixation and was then frozen. Urine was collected in a plastic bucket containing 10% (v/v) H_2_SO_4_ through the handmade urine bag. The total urine output was collected, measured, and weighed every morning. The urine from each calf that was collected over days 2 to 5 was mixed, and a small portion of the urine was analyzed for N and urine energy (UE) concentration.

### Chemical analysis

The DM content of each sample was determined after drying to constant weight in an oven at 65°C [18]. A bomb calorimeter (6400; PARR Instrument Co, Moline, IL, USA) was used to analyze the energy concentration in the forage, concentrate, feces, and urine. A 10 mL sample of urine from each calf was passed through quantitative filter paper, and then the filter paper was dried at 55°C for 30 minutes before the gross energy (GE) of the urine was measured [19]. The total N concentration was determined using a Kjeldahl nitrogen analyzer (Model K9840; Hanon Instruments, Jinan, China), using method 990.03, AOAC [20]. The feces were combusted at 550°C for 10 hours in a muffle furnace (SX-G30102; Shanghai Liangyi Scientific Instrument Co., Ltd, Shanghai, China), and the OM content of the grass was measured, following AOAC [18] method 942.05. Heat stable alpha amylase was added to the solution to wash the samples before the neutral detergent fibre (NDF) content was measured. Both the NDF content and the acid detergent fibre (ADF) content were determined using filter-bag technology [20] that was adapted for a semi-automatic fiber analyzer (A2000i; ANKOM Instrument Co., Ltd, Macedon, NY, USA). The NDF and ADF contents are expressed inclusive of residual ash. Ammonia-N was analyzed by phenol-hypochlorite colorimetric procedures [21].

Each indirect open-circuit respiration calorimeter chamber (length, 4.2 m, width, 1.95 m, height, 2.2 m) included an exhaust ventilation system, a measurement system, a temperature and humidity control system, and a sampling and analysis system. The exhaust air exchange system mainly adjusted the air exchange rate in the cabin and the number of air exchanges per hour through the main air outlet valve and the fan bypass inlet valve. Chambers were made of double-perspex and aluminum frames and equipped with airlocks. A slight negative pressure was applied to each chamber using a gas flow meter (GFM57; Aalborg, Orangeburg, NY, USA.) and a separate electric motor. The flow rates was set at approximately 50 to 55 m^3^/h. Temperature and humidity were controlled using humidity-sensing probes (HDC3020-Q1; Texas Instruments Inc., Dallas, TX, USA) and air conditioning devices (FCR7.2Pd/Ena; Zhuhai Gree Electric Appliance Co., Ltd. Zhuhai, China) that were set to maintain 22°C and 55% relative humidity. The CH_4_, CO_2_, and oxygen (O_2_) levels were measured for 3 minutes in each chamber when switching to that chamber using a multi-channel gas sampling instrument (YA-03DLQ; Yi’an Tech. Co. Ltd., Lanzhou, China) and a VA-3113 multi-function gas analyzer (Horiba Trading Co. Ltd., Beijing, China). The sampling pump suction rate of the multichannel gas sampling instrument was 0.5 L/min. Gas production, temperature and humidity were measured and analyzed in the bottom, middle and upper areas of each chamber. The gas was sampled at the end of the air outlet. The gas analyzer was composed of built-in sensor modules such as non-dispersive infrared absorptiometry (NDIR) and magnetic pressure analysis (MPA). The main detection components of NDIR were CO_2_ (Measuring range: Min, 0 to 200 ppm; Max, 0 vol% to 100 vol%) and CH_4_ (Measuring range: Min, 0 to 200 ppm; Max, 0 vol% to 100 vol%). The main detection components of MPA were O_2_ (Measuring range: Min, 0 vol% to 5 vol%; Max, 0 vol% to 25 vol%). The sampling and analysis system was calibrated before the start of each gas test using calibration gases, including N_2_ and 201.4×10^−6^ mol/mol CH_4_, 22.8% O_2_, and 2,014.4×10^−6^ mol/mol CO_2_ (Gases CO., LTD, Dalian, China) [7]. Known volumes of CH_4_, O_2_, and CO_2_ were released into the chamber at the beginning and end of the trial, and the recovery of these gases was measured. The main method of CH_4_ recovery of the flow measurement system was checked by releasing analytical grade CH_4_ into the chamber before and after the experiment. Calculation of A emissions: A emissions = the concentration differences of A between the air into and out of each chamber × the total volume of gas exchange; the total volume of gas exchange = flow rate × interval time; A is CH_4_, O_2_, and CO_2_.

### Calculation of the energy contents

The formulas used to calculate digestive energy intake (DEI), CH_4_ energy (CH_4_-E) output, metabolizable energy intake (MEI), heat production (HP), and retained energy (RE) are [22]:


$$\begin{array}{l} \text{DEI }(\text{MJ}/\text{d})=\text{GE intake }(\text{GEI},\text{MJ}/\text{kg})-\text{FE output }(\text{MJ}/\text{kg})\\ {\text{CH}}_{4}\text{-E output }(\text{MJ}/\text{d})={\text{CH}}_{4}\text{-E output }(\text{L}/\text{d})\times 39.54\;(\text{kJ}/\text{L})\times 10^{-3}\\ \text{MEI }(\text{MJ}/\text{d})=\text{DEI }(\text{MJ}/\text{d})-\text{UE Output }(\text{MJ}/\text{d})-{\text{CH}}_{4}\text{-E output }(\text{MJ}/\text{d})\\ \text{HP }(\text{MJ}/\text{kg})=[16.18\times {\text{O}}_{2}\;\text{consumption }(\text{L}/\text{d})+5.20\times {\text{CO}}_{2}\;\text{emission }(\text{L}/\text{d})-2.17\times {\text{CH}}_{4}\;\text{emission }(\text{MJ}/\text{d})-5.99\times \text{N excretion }(\text{urinary N},\text{g}/\text{d})]\times 10^{-3}\\ \text{RE }(\text{MJ}/\text{d})=\text{MEI }(\text{MJ}/\text{d})-\text{HP }(\text{MJ}/\text{d}) \end{array}$$

### Statistical analysis

All analyses were analyzed using SPSS 19.0 (Inst., Chicago, IL, USA). The homogeneity of variances was tested. The normality of the data was analyzed using the Shapiro-Wilk test. The data were analyzed using a the one-way analysis of variance. Regression analysis was used to examine the linear and quadratic effects of GS on nutrient intake, nutrient digestibility, Energy utilization, and CH_4_ emissions. Less than 0.05 p value was considered a statistically significant difference in the data, while 0.05<p<0.10 has a tend.

## RESULTS

### Performance, nutrient intake and digestibility

**Figure 1:**
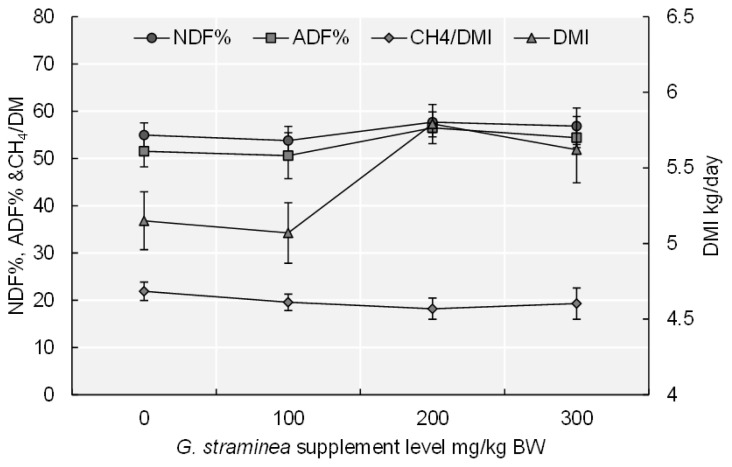
Sketch diagram of the correlationship between DMI (red closed bar), NDF% (green closed bar), ADF% (purple closed bar), enteric CH_4_ emission (bule closed bar) and the supplement level of GS. NDF%, neutral detergent fibre (NDF) digestibility; ADF% acid detergent fibre (ADF) digestibility; CH_4_/DM, methane (CH_4_) emissions/dry matter; DMI, dry matter intake; BW, body weight; GS, *Gentiana straminea*. Values are mean± standard error of the mean.

The basal diet intake and ADG were significantly (p<0.05) higher for the 200 GS and 300 GS treatments than for the other treatments (Table 2). The DMI in the 200 GS and 300 GS treatments was significantly higher (p<0.01) than 0 GS and 100 GS treatments (Figure 1). With increasing GS supplementation level, basal diet intake (p<0.05), ADG (p<0.05), and DMI (p<0.01) also revealed quadratic changes. The N intake increased significantly (p<0.05) as the amount of the GS-supplemented increased. Adding 200 mg/kg BW of GS to the basal diet increased the OM intake, relative to that of the 0 GS treatment (p<0.05) and to that of the other two supplementation treatments. ADF intake increased significantly when the basal diet was supplemented with 100 and 200 mg/kg BW of GS, compared with when the basal diet was provided with no supplement and when GS was supplemented at 300 mg/kg BW (p<0.01). Dietary GS supplementation resulted in quadratic increases of NDF (p<0.05) intake and ADF intake (p<0.05). The digestibility of DM was greater for the 100 GS treatment than for the 0 GS treatment (p<0.05). The digestibility of NDF (p<0.05), and ADF (p<0.05) was greater for the 200 GS and 300 GS treatments than for the 0 GS treatment, and the digestibility of ADF and NDF increased firstly and then decreased with the addition level of GS.

The effects of GS supplementation on N utilization are listed in Table 3. N retention increased (p<0.01) when GS was included in the diets, and the increase was significant for the 200 GS and 300 GS treatments relative to the 0 GS treatment (p<0.001). Supplementation with GS significantly raised retention of N relative to N intake (p<0.001). In contrast, there was a clear and obvious (p<0.05) reduction in urinary N relative to N intake when the diet was supplemented with GS. With increasing GS supplementation level, fecal N (p = 0.057) and retention N (p<0.05) also revealed quadratic changes.

### Energy utilization

Table 4 shows the impact of supplementing a calf’s diet with GS on energy efficiency. Supplementating the diet with GS resulted in no significant change to FE output. GEI and RE were enhanced (p<0.05 and p<0.001, respectively) when the diet was supplemented with GS. DEI and MEI were significantly (p<0.05) greater for the 100 GS and 200 GS treatments relative to the 0 GS. The UE output was significantly (p<0.05) greater for the treatments that received a diet supplemented with 200 and 300 mg/kg BW GS relation to the other treatments. With increasing GS supplementation level, GEI (p< 0.01) and FE output (p<0.001) also revealed quadratic changes. CH_4_-E output was significntly (p<0.05) lower for the treatments that received diets supplemented with GS than for the treatments that received only the basal diet. The HP increased significantly (p<0.05) in the treatments that receved a diet supplemented with 200 mg/kg BW GS, relative to the 0 GS treatment.

### CH**_4_** emissions

Compared with the 0 GS treatment, the average daily amount of CH_4_ produced by the calves decreased significantly (p< 0.001) when the diet was supplemented with GS (Table 5). CH_4_ emissions per kilogram of DMI were significantly (p< 0.05) lower for the 200 GS and 300 GS treatments than for the 0 GS treatment (Figure 1). CH_4_ per BW^0.75^ was significantly (p<0.05) lower for the treatments that received GS supplementation relative to the 0 GS treatment. Compared with the 0 GS treatment, a diet supplemented with 200 mg/kg BW of GS significantly reduced CH_4_/OM intake. Dietary GS addition resulted in a quadratic increase in the CH_4_ (p<0.01), CH_4_/BW^0.75^ (p = 0.078), and CH_4_/DMI (p<0.01).

## DISCUSSION

Supplementing the diet of mice with GS increases the total antioxidant capacity, and increases superoxide dismutase and glutathione peroxidase activity in the serum, liver, and muscles, enhancing the body’s anti-stress ability and indirectly promoting growth [23]. Experiments on mice have also shown that the smell of GS can stimulate eating and that GS can promote the secretion of gastric juices and pepsin activity, thereby improving the digestibility of nutrients [24]. The quadratic increases in ADG and DMI with supplementation in GS was observed in this study, which reached the peak of DMI and ADG at supplemental 200 and 300 mg/kg BW. In fact, dietary GS addition failed to modify DMI and ADG in the 100 GS. These results indicate that additional dosage above 100 mg/kg BW may be needed to examine the effects of dietary GS addition on ADG and DMI. This may at least partially explain why a diet supplemented with 200 and 300 mg/kg BW GS increased intake of the basal diet, DMI, NDF intake, DMD, and ADG and may also explain why a diet supplemented with GS may improve N intake (kg/d) and GEI. Flavonoids in plants may improve production performance, nutrient digestibility, and rumen fermentation [8], and flavonoids (3.87%) in GS may have similar effects. Ma et al [8] found that supplementing the diet of sheep with mulberry leaf flavonoids could increase dietary fiber utilization by promoting cellulolytic bacteria, thereby increasing NDF and ADF digestibility. Those findings are similar to the results from this study, in which the digestibility of NDF and ADF increased significantly in the treatment groups that received a diet supplemented with 100 and 200 mg/kg BW GS.

N retention reflects the protein state of ruminants [25]. In this study, N intake, N loss, and N retention were affected by GS supplementation, although GS did not influence N digestibility (Table 3). During the experiment, the calves in the treatments that received the supplemented diet were in a state of positive N utilization. These results are consistent with the effects of natural plant-based additives on growth performance and N utilization for ruminants, but they differ from the findings presented in Ma et al [8]. In that study, supplementing ruminant diets with plant extracts did not cause changes in N intake or in N retention. The increase in N intake in this study may be due to the increased feed intake. This study also observed that N intake (r^2^ = 0.040, p = 0.072) was positively correlated with GS supplementation. The GS may enhance retention N by promoting the secretion of penpsin activity [24], as retention N (r^2^ = 0.321, p = 0.022) increased linearly with GS supplementation. The reduction in urine N relative to N intake for the treatments that received diets supplemented with GS, as compared with the 0 GS treatment, shows that the supplement may be an effective way to eliminate environmental effects from volatile N excretion, because the ammonia produced from hydrolysis of urea is easily volatilized and lost from animal production to the environment [26]. In this study, fecal and urinary N output and N retention were linearly and positively related to N intake for the groups that received a diet supplemented with GS. Therefore, supplementing the diets of calves with GS may be a means of reducing volatile nitrogen emissions in the environment.

Plant additives affect ruminant feed intake and total energy intake due to their special active ingredients [27]. Urine, feces, and CH_4_ emissions are classified as lost energy [28]. In this study, GEI, DEI, and MEI were higher for the 100 and 200 mg/kg BW treatments than for the 0 GS treatment (Table 4). This can be explained by the increase in appetite and feed digestibility for these treatments, relative to the 0 GS treatment. The current study found that when HP increased for the 200 GS treatment, MEI also increased. This is consistent with previous studies, that have shown that an increase in HP is accompanied by an increase in MEI for hybrid beef cattle [29]. Injecting a GS solution into mice can speed up the gastric emptying rate and reduce the residence time for food in the stomach [24]; adding GS to the diet of Simmental calves may have had a similar effect. There were quadratic decreases in CH_4_ emission (g/d) with supplementation in GS observed in this study. The reductions in CH_4_ emissions, and in CH_4_ energy, may be due to the fact that feed stayed in the rumen for a shorter time in calves that received diets with GS. CH_4_ emissions and CH_4_-E production are negatively correlated with DMI and GEI [30]. This may explain the increases in total energy intake and DMI and the simultaneous decreases in CH_4_ emissions and CH_4_-E, for the treatments that received supplements of 200 and 300 mg/kg BW, relative to the 0 GS treatment. Compared with the 0 GS treatment, RE increased significantly in groups that received GS supplements, which may be attributable to the decrease in CH_4_-E for the groups that received the supplements. As a possible alternative mechanism for reducing CH_4_ emissions, Patra et al [31] found that propionic acid and butyric acid compete with methanogens in the rumen for the H^+^ that is required for CH_4_ production. The CH_4_ per unit DMI for the 200 and 300 mg/kg BW treatments decreased as the amount of GS supplementation increased. This is explained by the fact that one effect of GS is to accelerate emptying of the feed in the stomach, thereby reducing the time during which methanogenic microorganisms have access to the feed in the rumen for decomposition [31]. The quadratic decreases in CH_4_/DMI with supplementation in GS was observed in this study, which reached the peak of CH_4_/DMI at supplemental 200 and 300 mg/kg BW. In fact, dietary GS addition failed to modify CH_4_/DMI in the 100 GS. These results indicate that additional dosage above 100 mg/kg BW may be needed to examine the effects of dietary GS addition on CH_4_/DMI. CH_4_-E/GEI, CH_4_-E/DEI, and CH_4_-E/MEI were significantly reduced in the 200 GS and 300 GS treatments, relative to the 0 GS treatment,which may be due to the higher feed intake for the treatments. CH_4_ emissions were lower for the treatments that received GS supplementation. The anticipated benefits to energy utilization are one reason for using natural plants, instead of antibiotics and other additives, in ruminant production.

## CONCLUSION

Under the conditions described for this study, adding 100 mg or 200 mg supplements of GS to the diet of Simmental calves resulted in enhanced ADG, DMI, and nutrient digestibility. In addition, diet supplements of GS could improve N utilization, energy utilization, and reduce CH_4_ emissions, while having no negative effect on the rumen fermentation of calves. These findings confirm the effect of GS on biological activity, however a diet supplemented with GS also had beneficial effects animal growth, feed digestibility and energy utilization, making the supplement a good source for ruminant nutrition.

## Figures and Tables

**Table 1:** Chemical composition of the experimental diets (based on DM)

Nutrient level	Basal diet (%)	*G. straminea* (GS)(%)
DM	87.9	91.38
OM	89.36	83.37
CP	14.95	13.61
NDF	39.91	29.7
ADF	20.71	17.81
EE	1.62	0.29
Pre-mix^1)^	0.70	-

DM, dry matter; OM, organic matter; CP, crude protein; NDF, neutral detergent fibre; ADF, acid detergent fibre; EE, ether extract.

1) Manufactured by the Cangzhou Land Prataculture Center, Hebei, China. The pre-mix contained (per kg): 1×10^6^ IU vitamin A, 1×10^4^ IU vitamin D_3_, 500 IU vitamin E, 100 mg vitamin K_3_, 100 mg vitamin B_1_, 100 mg vitamin B_2_, 200 mg vitamin B_6_, 6 mg vitamin B_12_, 100 mg niacin, 200 mg calcium pantothenate, 1,000 mg methionine, 50 mg lysine.

**Table 2:** Effect of *Gentiana straminea* supplementation on nutrient intake and digestibility in Simmental calves

Item	Treatments^1)^				SEM	p-value		
	
	0 GS	100 GS	200 GS	300 GS		ANOVA	Linear	Quadratic
Basal diet intake (kg/d)	5.15^b^	5.05^b^	5.76^a^	5.57^a^	0.087	0.001	0.003	0.014
ADG (kg/d)	0.82^b^	0.78^b^	1.04^a^	0.95^a^	0.130	0.030	0.003	0.010
DMI (kg/d)	5.15^b^	5.07^b^	5.79^a^	5.62^a^	0.091	0.001	0.002	0.009
Nutrient intake (g/kg BW^0.75^)								
DMI	104.57	103.53	114.50	106.18	1.69	0.069	0.704	0.474
OM intake	92.91^b^	91.26^b^	100.20^a^	93.36^b^	1.280	0.045	0.226	0.080
NDF intake	45.37^c^	46.96^bc^	53.76^a^	52.37^ab^	1.210	0.016	0.009	0.026
ADF intake	24.84^b^	28.56^a^	27.55^a^	25.20^b^	0.463	0.001	0.801	0.024
N intake	2.50^b^	2.87^a^	2.85^a^	2.88^a^	0.063	0.032	0.061	0.072
Nutrient digestibility (%)								
DMD	63.29^a^	60.48^b^	64.98^a^	64.41^a^	0.006	0.040	0.208	0.412
OM digestibility	67.82	66.5	66.66	67.60	0.701	0.902	0.413	0.707
NDF digestibility	54.94^b^	53.78^b^	57.67^a^	56.82^a^	1.859	0.031	0.303	0.716
ADF digestibility	51.53^b^	50.58^b^	56.46^a^	54.38^a^	0.856	0.046	0.116	0.275
N digestibility	81.79	80.58	81.46	81.13	0.363	0.701	0.925	0.815

SEM, standard error of the mean; ANVOA, analysis of variance; ADG, average daily gain; DMI, dry matter intake; BW, body weight; OM, organic matter; NDF, neutral detergent fibre; ADF, acid detergent fibre; N, nitrogen; BW, body weight.

1) 0 GS, 0 mg/kg BW, the control; 100 GS, 100 mg/kg BW; 200 GS, 200 mg/kg BW; 300 GS, 300 mg/kg BW.

a–c Significant differences in the table (p<0.05).

**Table 3:** Effect of *Gentiana straminea* supplementation on N utilization in Simmental calves

Item	Treatments^1)^				SEM	p-value		
	
	0 GS	100 GS	200 GS	300 GS		ANOVA	Linear	Quadratic
N intake (kg/d)	0.14^c^	0.15^b^	0.16^a^	0.15^b^	0.001	<0.001	0.368	0.315
Fecal N (kg/d)	0.050	0.054	0.055	0.052	0.002	0.691	0.593	0.057
Urinary N (kg/d)	0.070	0.063	0.069	0.064	0.002	0.842	0.634	0.881
Retention N (kg/d)	0.021^b^	0.028^a^	0.032^a^	0.032^a^	0.006	<0.001	0.001	0.022
Ammonia-N (mmol/L)	10.67	10.35	11.49	10.66	0.315	0.637	0.351	0.651
Fecal N/N intake (kg/kg)	0.35	0.38	0.35	0.35	0.013	0.87	0.741	0.775
Urinary N/N intake (kg/kg)	0.50^a^	0.42^b^	0.44^b^	0.43^b^	0.009	0.01	0.318	0.468
Retention N/N intake (kg/kg)	0.15^b^	0.19^a^	0.20^a^	0.21^a^	0.001	<0.001	0.068	0.148

SEM, standard error of the mean; ANOVA, analysis of variance.

1) 0 GS, 0 mg/kg BW, the control; 100 GS, 100 mg/kg BW; 200 GS, 200 mg/kg BW; 300 GS, 300 mg/kg BW.

a–c Different letters represent significant differences in the table (p<0.05).

**Table 4:** Effect of *Gentiana straminea* supplementation on energy utilization in Simmental calves

Item	Treatments^1)^				SEM	p-value		
	
	0 GS	100 GS	200 GS	300 GS		ANOVA	Linear	Quadratic
GEI (MJ/kg BW^0.75^)	1.99^b^	2.04^a^	2.06^a^	2.05^a^	0.018	0.042	0.170	0.005
FE output (MJ/kg BW^0.75^)	0.71	0.72	0.74	0.77	0.034	0.430	0.024	<0.001
UE output (MJ/kg BW^0.75^)	0.04^b^	0.04^b^	0.05^a^	0.05^a^	0.002	0.029	0.106	0.447
DEI (MJ/kg BW^0.75^/d)	1.28^b^	1.32^a^	1.31^a^	1.28^b^	0.045	0.033	0.937	0.189
MEI (MJ/kg BW^0.75^/d)	1.10^b^	1.16^a^	1.23^a^	1.10^b^	0.029	0.023	0.854	0.437
HP (MJ/kg BW^0.75^/d)	0.10^b^	0.11^ab^	0.12^a^	0.11^ab^	0.003	0.032	0.398	0.372
RE (MJ/kg BW^0.75^/d)	0.90^b^	1.05^a^	1.01^a^	1.00^a^	0.031	0.006	0.474	0.446

SEM, standard error of the mean; ANOVA, analysis of variance; GEI, gross energy intake; FE, fecal energy; UE, urine energy; DEI, digestive energy intake; MEI, metabolizable energy intake; HP, heat production; RE, retained energy.

1) 0 GS, 0 mg/kg BW, the control; 100 GS, 100 mg/kg BW; 200 GS, 200 mg/kg BW; 300 GS, 300 mg/kg BW.

a,b Different letters represent significant differences in the table (p<0.05).

**Table 5:** Influence of dietary *Gentiana straminea* supplementation on methane emission in Simmental calves

Item	Treaments^1)^				SEM	p-value		
	
	0 GS	100 GS	200 GS	300 GS		ANOVA	Linear	Quadratic
CH_4_ (g/d)	126.17^a^	114.26^b^	112.98^b^	117.04^b^	1.523	0.004	0.063	0.003
CH_4_/BW^0.75^ (g/kg)	2.71^a^	2.42^b^	2.37^b^	2.47^b^	0.053	0.041	0.341	0.078
CH_4_/DMI (g/kg)	21.88^a^	19.56^a^	18.20^b^	19.27^b^	0.449	0.022	0.072	0.004
CH_4_/OMI (g/kg)	33.28^a^	30.43^ab^	27.54^b^	30.03^ab^	0.662	0.024	0.506	0.334

SEM, standard error of the mean; ANOVA, analysis of variance.

1) 0 GS, 0 mg/kg BW, the control; 100 GS, 100 mg/kg BW; 200 GS, 200 mg/kg BW; 300 GS, 300 mg/kg BW.

a,b Different letters represent significant differences in the table (p<0.05).
